# Interactive Drivers of Activity in a Free-Ranging Estuarine Predator

**DOI:** 10.1371/journal.pone.0080962

**Published:** 2013-11-18

**Authors:** Matthew D. Taylor, Luke McPhan, Dylan E. van der Meulen, Charles A. Gray, Nicholas L. Payne

**Affiliations:** 1 Port Stephens Fisheries Institute, Fisheries NSW, Taylors Beach, New South Wales, Australia; 2 School of Biological, Earth and Environmental Science, University of New South Wales, Sydney, New South Wales, Australia; 3 Batemans Bay Fisheries Centre, Fisheries NSW, Batemans Bay, New South Wales, Australia; University of Hamburg, Germany

## Abstract

Animal activity patterns evolve as an optimal balance between energy use, energy acquisition, and predation risk, so understanding how animals partition activity relative to extrinsic environmental fluctuations is central to understanding their ecology, biology and physiology. Here we use accelerometry to examine the degree to which activity patterns of an estuarine teleost predator are driven by a series of rhythmic and arrhythmic environmental fluctuations. We implanted free-ranging bream *Acanthopagrus australis* with acoustic transmitters that measured bi-axial acceleration and pressure (depth), and simultaneously monitored a series of environmental variables (photosynthetically active radiation, tidal height, temperature, turbidity, and lunar phase) for a period of approximately four months. Linear modeling showed an interaction between fish activity, light level and tidal height; with activity rates also negatively correlated with fish depth. These patterns highlight the relatively-complex trade-offs that are required to persist in highly variable environments. This study demonstrates how novel acoustic sensor tags can reveal interactive links between environmental cycles and animal behavior.

## Introduction

From a history of biochronological study, we can derive several overarching relationships which are relatively consistent among animals: 1) photoperiodic cycles are the most powerful entraining agent of circadian rhythms; 2) intra- and inter-specific interactions contribute to this behavioral rhythmicity [1]; and, 3) rhythms in behavior are often associated with exogenous processes over other scales [2]. Consequently, the ability to measure and characterize activity patterns is central to our understanding of the physiological, ecological and social aspects of natural systems. Inter-specific variation in rhythmicity and interactions with other organisms are tightly linked; playing a fundamental role in shaping the evolution of natural systems [3].

Rhythmic behaviors often represent traits which have evolved in response to many biotic factors including predation risk [4], [5], food availability and quality [5], and abiotic influences such as light and tide (over short time periods), and temperature and turbidity (over longer time periods). For example, adaptive foraging strategies and habitat use mediated by diel rhythms can facilitate sympatry by partitioning access to resources [6]. Further, minimizing predation risk whilst optimizing energetic gain is often achieved by organisms through behaviours linked to exogenous rhythms [7], [8]. A common strategy for some fishes involves increased activity related to foraging at night, which facilitates both avoidance of visual predators and increased access to nocturnally active prey [9]. In understanding the links between external influences and behaviour, it is important to concurrently consider multiple entraining sources, and interacting factors that affect an organism's choice or strategy (e.g. prey quality, predation risk and habitat).

Many studies in aquatic systems have investigated behavioral rhythmicity at the diel scale. Examples include diel vertical migration in pelagic fish and krill [10]–[12], photoperiod entrained activity patterns in freshwater fish [13], and diel patterns in habitat usage in coral reef fish [14]. Aquatic systems, however, present a series of agents that may entrain behavioral rhythms, some of which are not experienced in the terrestrial sphere. One such example is tidal exchange; a sub-diel rhythm occurring in marine and estuarine environments approximately twice over a single diel period in some parts of the world [15], [16], the strength of which is also influenced by lunar cycles. Consequently, factors co-occurring at different frequencies in aquatic systems can lead to concomitant effects on the behavioral patterns which are entrained to them [17]. This is particularly evident in estuarine environments, where tidal cycles can greatly affect both ambient hydrographic conditions [18] and connectivity with particular habitats [19]. Thus, the interactive effects of multiple exogenous factors may have important implications for understanding partitioning of time and space at the species level, and inter-specific interactions at the ecosystem level.

Studying the activity patterns of mobile aquatic animals in their natural habitat is logistically difficult. This is principally due to the difficulties associated with observing individuals underwater, at night [20], [21], in turbid environments and at depths which cannot be readily accessed for observation [22]. Telemetry approaches have helped to address these difficulties, and modern technology facilitates the simultaneous collection of space use, movement and abiotic information (e.g. temperature and depth) at fine temporal scales [23], [24]. Recent advances have seen the inclusion of accelerometer sensors alongside acoustic transmitters within implantable tags. These can measure, log and transmit acceleration data to acoustic receivers without a need for retrieval, and have emerged as a powerful tool for studying activity rates of free-ranging aquatic organisms [7], [25], [26].

This study used accelerometry with the broad objective of understanding activity rhythms in a species inhabiting a highly variable estuarine environment. Specifically, we used bream *Acanthopagrus australis* as a model to test for the interactive effects of diel, tidal and lunar rhythms on activity patterns, alongside several arrhythmic fluctuations. *Acanthopagrus australis* is a sparid endemic to the east coast of Australia, and spend the majority of their lifecycle in estuaries and inshore coastal waters [27]. The species feeds primarily on molluscs, polychaetes and crustaceans [28], and are thought to exploit bare substrates, seagrass beds and mangroves as foraging habitats [29].

## Materials and Methods

### Study site and acoustic array

**Figure 1:**
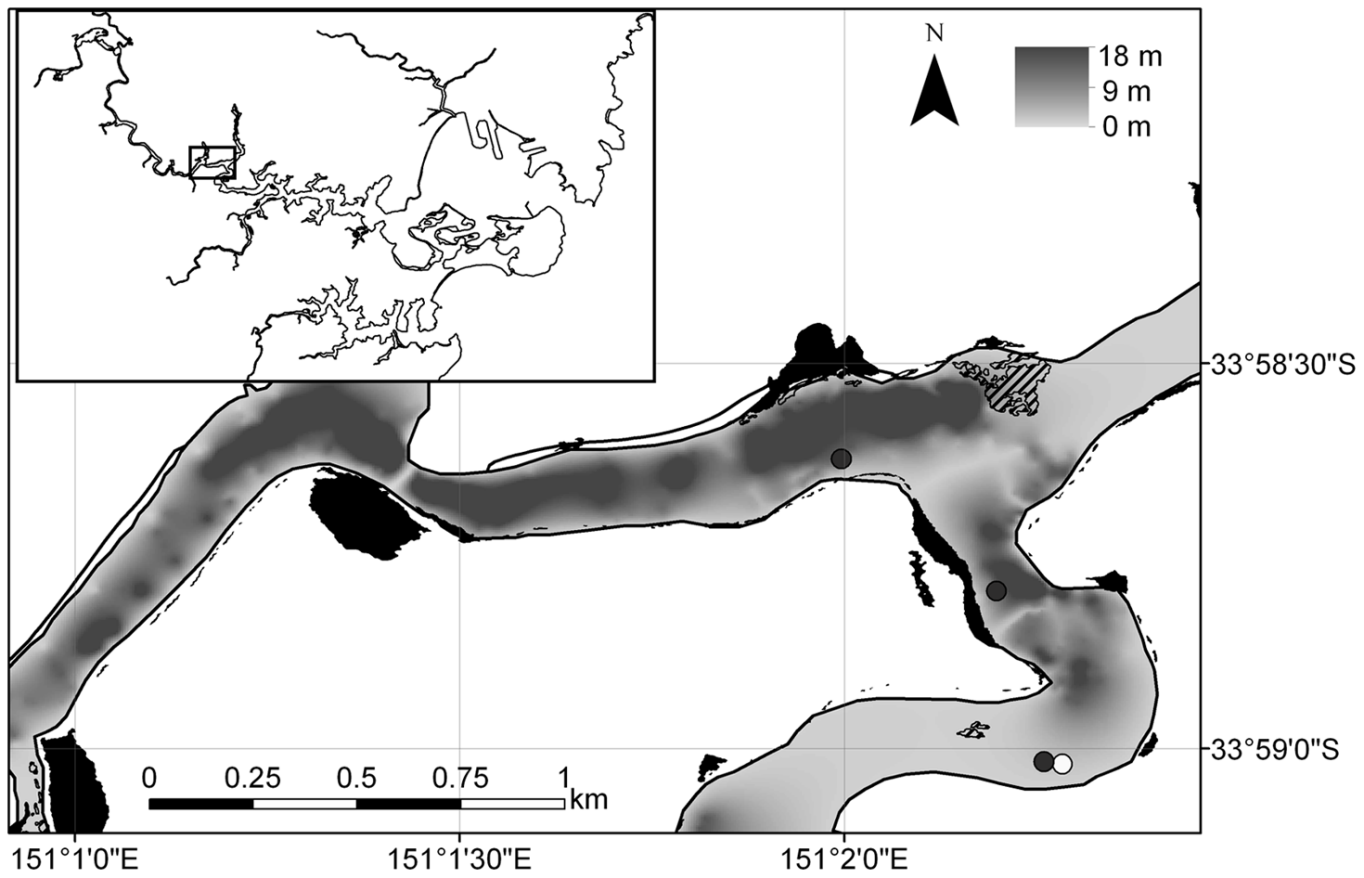
Map of the study area in the Georges River, southern Sydney, showing receiver locations (black circles), the Botany Bay Water Quality Improvement Program logger (white circle), *Zostera* sp. seagrass beds (hatched polygons), and mangroves (black polygons). A bathymetry surface is also shown as a gradient between 0(shown in Figure legend in top right).

The study was conducted in the Georges River, in the southern Sydney metropolitan area (34.008°S, 151.119°E). The river is 96 km long and drains a catchment of 960 km^2^, with the upper limit to the estuary being the Liverpool Weir 45 km upstream. The Georges River estuary is primarily urbanised [30], [31], and has experienced substantial shoreline modification and heavy urban pollution inputs. The Georges River contains limited saltmarsh habitat, but the mid-estuary contains considerable mangroves and small mangrove lined tributaries adjacent to the Georges River National Park. Fish were captured and tagged in this study in the vicinity of this area, at approximately 33.977° S 151.036° E (Fig. 1). An array of 34 Vemco VR2W acoustic receivers (Vemco, Halifax, Nova Scotia) was deployed throughout the entire estuary, and data from this array is presented elsewhere [7]. Here, we confined our dataset to acceleration (hereafter acceleration is used to refer to the data collected by the tags, and activity is used to refer to the behaviour that the data is describing) and depth measurements collected at the three receivers near Picnic Point in the Georges River, such that activity and depth data could be correlated to the explanatory variables collected at that location (see below, Fig. 1). The range of VR2W receivers is approximately 300 m [21], [32], [33], so these three receivers were spaced approximately 500 m apart, thus collecting data for approximately 1.5 km of river.

### Accelerometry transmitters and tagging

As accelerometry transmitters represent a relatively new technology, a brief introduction into the technical aspects of the tags is provided here. The accelerometer in Vemco tags can be programmed to collect either bi-axial or tri-axial acceleration data, with a user-specified sampling window and range of transmittable measurements. The acceleration sensor has a measurable range of ±29.4 m s^−2^ on each axis, and these values are used to calculate a composite root mean square acceleration estimate from the selected axes. The resultant vector is transmitted acoustically to the VR2W receiver, in the range 0–3.4 m s^−2^ (tri-axial accelerometer) or 0–4.9 m s^−2^ (bi-axial accelerometer). Any vectors greater than these ranges are transmitted as the maximum value (for example, a RMS estimate from a tri-axial accelerometer of 5.2 m s^−2^ will be transmitted as 3.4 m s^−2^). Tri-axial accelerometer measurements represent a general activity index of the fish; including surge (forward-backward) motion, dorsoventral movement, and lateral movement components, measured at 5 or 10 Hz on each axis. Bi-axial accelerometers measure two axes (dorsoventral movement and lateral movement), usually at a higher frequencies (10 Hz). Tri-axial accelerometers are analogous to overall dynamic body acceleration (ODBA [34], [35]), whereas bi-axial are analogous to partial dynamic body acceleration (PDBA [36]). As surge represents lower sustained acceleration as opposed to the dorsoventral and lateral undulations of a swimming fish, tri-axial vectors will usually be smaller than biaxial vectors. This coupled with greater frequency of measurement mean bi-axial vectors represent a more precise reflection of tail beat patterns or swimming speed.

The length of the sampling window for the acceleration sensor represents a compromise between battery life and ability to sample a variety of behaviors. The data collected over each sampling window likely reflects a variety of different behaviors, which are averaged out over the specified time. Consequently, with a longer sampling window, there is a greater chance of sampling a signal which corresponds to a discrete behavior, but that signal will be diluted by the remainder of the sampling window. Conversely, a shorter sampling window will provide a more precise estimate of the acceleration vector that represents a certain behavior, but because a smaller proportion of each cycle is sampled for acceleration the chance of actually detecting the behavior is lower.

Accelerometry transmitters used in this study were configured from the Vemco V9 range of sensor tags. We selected Vemco V9AP-2L accelerometry and pressure transmitters (69 kHz, 3.3 g in water, 66 mm length), with the accelerometer programmed to measure bi-axial (dorsoventral movement and lateral) acceleration at 10 Hz, over a sampling window of 33 s.

This study was carried out in strict accordance with the recommendations in *A Guide to Acceptable Procedures and Practices for Aquaculture and Fisheries Research*, 3^rd^ Edition [37]. The protocol was approved by the Animal Care and Ethics Committee of both the University of NSW (Permit number 11/30A) and the NSW Department of Primary Industries (Permit number 09/01). All surgery was performed under anesthesia, and all efforts were made to minimize suffering. Capture and tagging of fish in the Georges River during this study was permitted under Section 37 of the NSW *Fisheries Management Act 1994*, through Scientific Research Permit number P03/0086 (issued by NSW Department of Primary Industries). Six *Acanthopagrus australis* (338–687 g) (hereafter called bream) were angled at Picnic Point in the Georges River in NSW, Australia in late May 2011 (Fig. 1). Transmitters were fitted internally using conventional surgical techniques [7], [32]. The transmitter was placed within the body cavity such that the longest dimension was anteriorly-posteriorly oriented. Fish were held in an onboard tank until the effects of anesthetic were no longer evident, and then released back into the water at the site of capture. We anticipated minimal effects of tagging on fish behavior, and that these would be limited to the first days post-tagging (as has previously been found for bream [38]).

### Environmental variables

Environmental parameters (temperature [°C], turbidity [nephelometric turbidity units], and photosynthetically active radiation [PAR]) were recorded by a subsurface logger operated by the Botany Bay Water Quality Improvement Program at Picnic Point (Fig. 1). Lunar phase data (calculated daily on a scale of 0 =  new, 1 =  full) was obtained from Geoscience Australia (http://www.ga.gov.au/) and tidal heights (m) were collected from the Picnic Point tidal gauge (33.982°S, 151.000°E) operated by the Manly Hydraulics Laboratory (www.mhl.nsw.gov.au).

### Statistical analysis

Raw data produced in this study are stored in the Australian Animal Tracking and Monitoring Systems e-Marine Infrastructure Initiative Database (http://aatams.emii.org.au/aatams/). Depth (pressure) values were adjusted to Indian Spring Low Water (ISLW) height datum on tidal height at the time the sensor measurement was taken (approach described in [9]), and thus represent a relative measure of fish depth. Mean values for all variables (bream activity, bream depth, temperature, turbidity, PAR, tide height and lunar phase) were analysed as 15 min time bins for each individual across the monitoring period (25/5/2011 to 11/9/11), and a series of generalized linear models were constructed to identify the dominant drivers of bream activity. Since our main hypothesis involved the interactive effects of circadian and ultradian rhythms common in the estuarine environment on activity patterns, we included a *PAR·Tide* interaction term in the model. The full model was thus of the form:
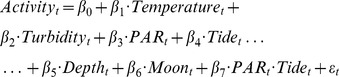
where independent variables reflect those described earlier in the methods. To account for potential serial correlation in our data, we fitted models using generalized least squares and modeled the autocorrelation between successive data points following the approach described in [39]. Several models were run using the gls function in R v. 2.12.1 (using the Linear and Nonlinear Mixed-effects Models package [40]), to determine the type (auto-regressive [AR], moving average [MA], or both autoregressive and moving average [ARMA]) and order of the error structure which best described autocorrelation in our data, on the basis of Akaike Information Criteria (AIC [41]). This was then incorporated into models constructed to determine the best combination of explanatory variables and their relative contributions to bream activity, using the gls function in conjunction with the stepAIC routine [42]. Activity data were log-transformed to achieve a Gaussian distribution, predictors were standardized following Aitken & West [43] and assessed on the basis of Akaike's Information Criterion (AIC), with terms eliminated from the model if their removal increased the AIC (ΔAIC) by less than 2. All analyses were performed using R v. 2.12.1 (R-Core Development Team).

## Results

**Figure 2:**
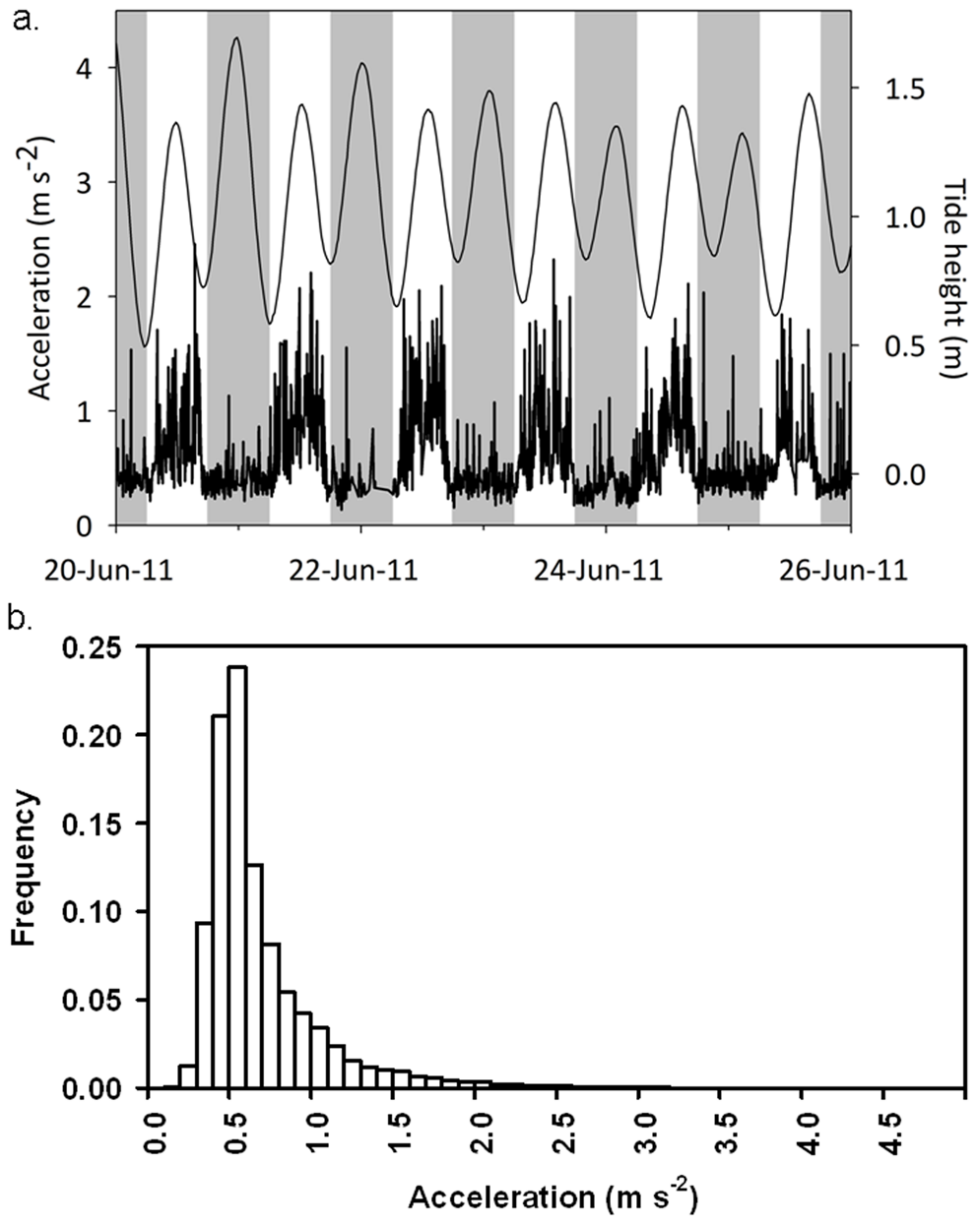
Actogram representative of fish activity (acceleration) across diel and tidal periods (a; data are for fish 3 in Table 1, between the dates 20^th^ June 2011 and 26^th^ June 2011), and frequency histogram of acceleration values measured in the study (b). Tidal height (a; secondary *y-*axis) is represented by the solid black line, and grey shading indicates night and white shading indicates day.

A total of 37,070 acceleration values and 37,066 depth values were recorded from the six individuals (Table 1), which resulted in data for 15,014 and 15,091 15-min bins of acceleration and depth, respectively. Fish displayed substantial circadian rhythmicity, as well as some evidence for elevated activity rates associated with peaks in the tidal period (Fig. 2a). Acceleration values ranged between 0.2 and 4.0 m s^−2^, with the majority of values ranging between 0.2 and 1.0 m s^−2^ (Fig. 2b).

**Table 1:** Tagging information for yellowfin bream (*Acanthopagrus australis*) tracked in the Georges River, showing the numbers of days on which data were recorded during the study period, the temporal window within which these data were recorded (e.g. for Fish 1, data were recorded on 89 days within a window of 100 d), and the number of activity and depth measurements from each tag recorded by the receivers shown in Figure 1.

Fish	Date tagged	Fork length (cm)	# days of data	Period (d)	Activity data	Depth data
1	16-05-11	28.0	89	100	11669	11785
2	17-05-11	24.0	49	54	7074	6981
3	19-05-11	30.0	92	110	17247	17202
4	02-06-11	30.4	7	58	196	203
5	02-06-11	27.9	11	76	408	423
6	02-06-11	26.0	11	66	476	472

Visual assessment of the autocorrelation function showed some correlation between the residual of each data point and multiple preceding data points, indicating autocorrelation was present in the data. Model results for the evaluation of autocorrelation are presented in Table 2; a first-order auto-regressive correlation structure provided the lowest AIC (1772.993), and was selected for further evaluation of the full model. Moving average and larger-order correlation structures were unnecessary as they failed to improve the AIC [39], [44], and the first-order auto-regressive function accounted for most of the correlation between time lags.

**Table 2:** Optimisation of the autocorrelation function, showing the auto-regressive (AR, *φ*_n_) and moving-average (MA, *θ*_n_) correlation parameters for models of increasing order.

AR order (*p*)	MA order (*q*)	*φ*_1_	*φ*_2_	*θ*_1_	AIC
0	0				1969.509
**1**	**0**	**0.17668**			**1772.993**
1	1	−0.07139		0.29979	1774.255
2	0	0.28949	−0.14868		1773.324

The AIC for each model was used to select the model which best described the error structure (shown in **bold**). As increasing complexity failed to produce models with a lower AIC, models with more than 2 auto-regressive and 1 moving average parameters were not run [39], [44].

**Figure 3:**
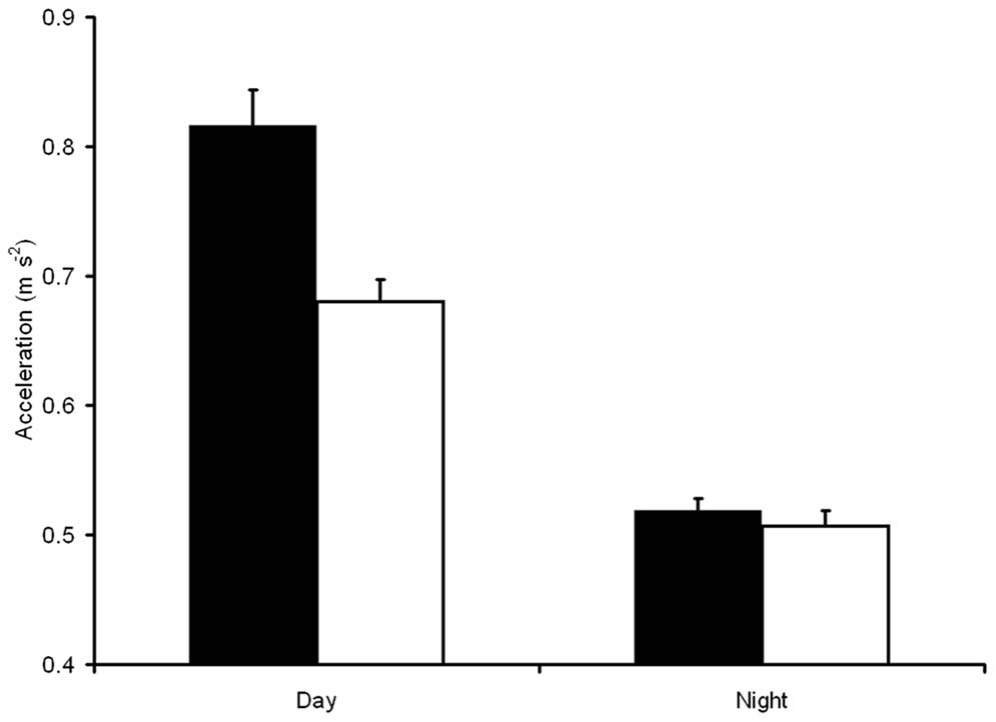
Categorical representation of acceleration across diel and tidal periods, showing greater activity rates during the day, and a clear peak in activity during the daytime high tide.

The series of model runs to determine the combination of independent variables which best explained variation in activity is displayed in Table 3. Variables which were sequentially removed from the model were *Lunar phase* and *Turbidity*, followed by *Temperature*. The most parsimonious model included the terms *PAR*, *Tide*, *Depth* and *PAR·Tide*; and the *φ* value indicated that the residuals separated by one time step had a correlation of 0.34. The parameter coefficients and tests for the significance of retained parameters are shown in Table 4. Comparison of parameter coefficients indicated that *Tide* and *PAR* were important parameters in driving activity values; however, the effect was not consistent across the diel period (as seen in the *PAR·Tide* interaction term). Evaluation of the *PAR·Tide* interaction term showed that the lowest activity values were at night regardless of tide, and the highest activity values were found during the day on a high tide (Fig. 3). There were no water quality variables (*Temperature* or *Turbidity*) identified in the best model, indicating that in our data set these were not important in driving activity patterns of bream.

**Table 3:** Sequential model runs using a first-order auto-correlation structure, and corresponding difference in AIC values.

Model	Parameters	Term removed	ΔAIC
Activity_p1q0_1	*Temperature* + *Turbidity* + *PAR* + *Tide* + *Moon* + *Depth* + *PAR·Tide*	*Moon* (P = 0.47)	3.41
Activity_p1q0_2	*Temperature* + *Turbidity* + *PAR* + *Tide* + *Depth* + *PAR·Tide*	*Turbidity* (P = 0.34)	1.92
Activity_p1q0_3	*Temperature* + *PAR* + *Tide* + *Depth* + *PAR·Tide*	*Temperature* (P = 0.28)	0.82
**Activity_p1q0_4**	***PAR*** ** + ** ***Tide*** ** + ** ***Depth*** ** + ** ***PAR·Tide***	*Depth* (P<0.01)	**0**
Activity_p1q0_5	*PAR* + *Tide* + *Depth*		6.05

The P-value of the term removed from each model is given, and the best model is highlighted in **bold**.

**Table 4:** Predictors from the most parsimonious auto-regressive model and their respective *β*-values, P-values for the response variable Activity (‘+’ *β* denotes a positive correlation and a ‘–’ *β* denotes a negative correlation).

Predictor	*β*	S.E.	*t*-value	p-value
*Diel period*	0.1651113	0.019	8.46	<0.001
*Tidal period*	0.1785145	0.035	5.01	<0.001
*Depth*	−0.0972872	0.029	−3.28	0.001
*Diel·Tide*	0.1120134	0.039	2.83	0.005
*φ*	0.34			

## Discussion

The influence of multiple extrinsic ecological variables on animal activity patterns is not well understood [45], [46], probably due to complexities associated with quantifying the various factors that can influence activity [46], [47]. This is exacerbated in aquatic habitats for two reasons: direct observation is logistically difficult; and many aquatic habitats are also characterized by the potentially strong influence of tides. While endogenous rhythms are most-often identified at the diel scale, there are several examples of aquatic animals exhibiting endogenous tidal rhythms, and these can persist in non-tidal environments [48], [49]. How then, should animals exposed to strong environmental fluctuations at multiple scales partition activity? Acceleration measurements of free-ranging bream indicate an interaction between exogenous fluctuations at the diel and tidal scale, with highest activity rates occurring during daytime high-tides. Diel-tidal interactions in the marine literature have been described for zooplankton behavior (e.g. [50]), but examples demonstrating concurrent entrainment to multiple rhythms in free-ranging fish are rare. Green jobfish *Aprion virescens*, displayed diel and tidal entrained habitat shifts, possibly between feeding and refuge habitats [51]; however these conclusions were based on detection frequency so may not represent actual patterns particularly well [21]. High inundation during the daytime provided the best foraging conditions for the surface-swimming four-eyed fish *Anableps anableps*, as visual predation was important for capture of insects [52]. In a study using pop-up archival transmitting (PAT) tags, basking sharks *Cetorhinus maximus* diving behavior was entrained to both diel and tidal rhythms, thought to facilitate exploitation of tidally-entrained aggregations of zooplankton prey at depth [53]. In a laboratory setting, endogenous circatidal and circadian components of fish movements have been shown to persist even in the absence of exogenous tidal cues, for an intertidal blenny (*Zoarces viviparous*) [54]. Finally, data collected from free-ranging North Sea plaice (*Pleuronectes platessa*) [55] equipped with electronic archival tags indicated that circatidal movement underlying selective tidal stream transport was modulated by diel periodicity [55].

Accelerometry and depth tags provided several lines of evidence, which taken together point to potential foraging behavior by bream over shallower intertidal areas when inundated during high tides. Bream are a visual, benthic predator [56] that primarily prey on polychaetes, bivalves and gastropods [28]. Higher activity rates coincided with individuals being present at shallow depths during the day. Habitat mapping of the study areas shows these shallow depths likely correspond to sand banks and mangroves where these types of prey species may be found at high densities. Lower nocturnal activity rates, and generally lower activity rates in deeper water, may reflect individuals resting and potentially hiding in deeper waters during the evening while larger estuarine predators are foraging in shallower waters (e.g. [9]). Mulloway (*Argyrosomus japonicus*) [57] and bull sharks (*Carcharinus leucas*) are two predators of bream and are nocturnally active [9], [58]; larger mulloway in particular concentrate foraging over shallow areas of estuaries at night, and are resident in deeper holes within the river during daytime [9]. Opposing diel rhythmicity of bream and its predators may allow bream to exploit their optimal foraging habitat whilst minimising exposure to predation. If our hypothesis about shallow intertidal foraging and resting in adjacent deeper waters is correct, these findings are largely consistent with foraging arena theory mediated by diel and tidal rhythmicity. Foraging arena theory maintains that spatial adjacency of foraging and resting habitat enhances energetic gains by minimizing the energetic cost and predation risk associated with accessing foraging areas from resting habitats [59]. Such temporal partitioning of habitat use is evident in a diversity of organisms, and adds to the growing body of examples which describe use of foraging arenas in aquatic systems (see [59] for a full review). This behaviour ultimately facilitates dynamic stability in aquatic systems, and description of these processes is essential for both the understanding of trophic flow of energy through such systems, and improving the design and parameterisation of widely used ecosystem models [60].

Lunar phase, temperature and turbidity are thought to affect bream activity, but had no detectable impact alongside the main entraining factors of diel and tidal cycles in our data set. In terms of their distribution along the estuary, bream are highly tolerant of minor environmental fluctuations [61], [62], although recent work indicates that a severe change in water quality is likely to have an influence on activity rates [7]. Temperature was largely invariant, and turbidity was generally invariant and low (∼6 ntu) during the study period, which may explain the lack of any impact on activity values during our study.

Any overarching influence of lunar phase on bream may not have been evident due to the time periods examined in our study. The effect of lunar phase on animal behaviour is most pronounced during spawning periods [16]; however bream are thought to spawn during winter in the northern section of their geographic range (spawning behaviour is unknown in the southern part of their range, where this study was conducted [29]). Considering all tagged fish in our study were at a size greater than the size at maturity, we did expect to observe some effect of lunar phase in our study if spawning was entrained to the lunar cycle. Given the limited temporal and spatial scale of the study, longer term monitoring is required to draw a firm conclusion on this hypothesis.

## Conclusion

This study demonstrates the utility of accelerometry tags for understanding complex rhythmic behavior of aquatic animals. For bream, multiple exogenous drivers (diel and tidal rhythmicity) interactively contributed to observed patterns of activity. Whilst this has allowed us to construct hypotheses regarding habitat use, resource partitioning and predation risk, future work should directly explore these links through simultaneous monitoring of activity, depth, habitat use (which could be achieved by deploying accelerometry tags within high resolution acoustic positioning systems [23]) and potentially, prey consumption. Such detailed knowledge of factors driving species behavior will greatly improve our understanding of the maintenance of dynamic stability in aquatic systems.

## References

[1] Favreau A, Richard-Yris M-A, Bertin A, Houdelier C, Lumineau S (2009) Social influences on circadian behavioural rhythms in vertebrates. An Behav 77: 983–989.

[2] Refinetti R, Menaker M (1992) The circadian-rhythm of body-termperature. Physiol Behav 51: 613–637. 1523238 10.1016/0031-9384(92)90188-8

[3] Yerushalmi S, Green RM (2009) Evidence for the adaptive significance of circadian rhythms. Ecol Lett 12: 970–981. 19566794 10.1111/j.1461-0248.2009.01343.x

[4] Berger D, Gotthard K (2008) Time stress, predation risk and diurnal-nocturnal foraging trade-offs in larval prey. Behav Ecol Sociobiol 62: 1655–1663.

[5] Zoufal R, Taborsky M (1991) Fish foraging periodicity correlates with daily changes of diet quality. Mar Biol 108: 193–196.

[6] Taylor MD (2008) Spatial and temporal patterns of habitat use by three estuarine species of mysid shrimp. Mar Freshw Res 59: 792–798.

[7] Payne NL, van der Meulen DE, Gannon R, Semmens JM, Suthers IM, et al (2013) Rain reverses diel activity rhythms in an estuarine teleost. Proc Roy Soc Ser B - Biol Sc 280: 20122363 http://dx.doi.org/20122310.20121098/rspb.20122012.20122363. 10.1098/rspb.2012.2363 PMC3574447 23173211

[8] Taylor MD, Ko A (2011) Monitoring acoustically tagged king prawns *Penaeus* (*Melicertus*) *plebejus* in an estuarine lagoon. Mar Biol 158: 835–844.

[9] Taylor MD, Laffan SD, Fielder DS, Suthers IM (2006) Key habitat and home range of mulloway (*Argyrosomus japonicus*) in a south-east Australian estuary: Finding the estuarine niche to optimise stocking. Mar Ecol Prog Ser 328: 237–247.

[10] de Pontual H, Jolivet A, Bertignac M, Fablet R (2012) Diel vertical migration of European hake *Merluccius merluccius* and associated temperature histories: insights from a pilot data-storage tagging (DST) experiment. J Fish Biol 81: 728–734. 22803732 10.1111/j.1095-8649.2012.03345.x

[11] Gibbons MJ (1993) Vertical migration and feeding of *Euphausia lucens* at 72 h stations in the southern Bengula upwelling region. Mar Biol 116: 257–268.

[12] Sims DW, Southall EJ, Tarling GA, Metcalfe JD (2005) Habitat-specific normal and reverse diel vertical migration in the plankton-feeding basking shark. J An Ecol 74: 755–761.

[13] Baktoft H, Aarestrup K, Berg S, Boel M, Jacobsen L, et al (2012) Seasonal and diel effects on the activity of northern pike studied by high-resolution positional telemetry. Ecol Freshw Fish 21: 386–394.

[14] Boaden AE, Kingsford MJ (2012) Diel behaviour and trophic ecology of *Scolopsis bilineatus* (Nemipteridae). Coral Reefs 31: 871–883.

[15] Raffaelli DG, Hawkins SJ (1999) Intertidal Ecology (2nd Edition). London: Kluwer Academic Publishers. 356 p.

[16] Krumme U (2009) Diel and Tidal Movements by Fish and Decapods Linking Tropical Coastal Ecosystems. In: Nagelkerken I, editor. Ecological Connectivity among Tropical Coastal Ecosystems. Dordrecht, NLD: Springer. pp. 271–324.

[17] Forward RB, Cronin TW (1980) Tidal rhythms of activity and phototaxis of an estuarine crab larva. Biol Bull 158: 295–303.

[18] Cronin TW, Forward RB (1979) Tidal vertical migration: An endogenous rhythm in estuarine crab larvae. Science 205: 1020–1022. 17795563 10.1126/science.205.4410.1020

[19] West JM, Zedler JB (2000) Marsh-creek connectivity: Fish use of a tidal salt marsh in Southern California. Est 23: 699–710.

[20] Harvey ES, Butler JJ, McLean DL, Shand J (2012) Contrasting habitat use of diurnal and nocturnal fish assemblages in temperate Western Australia. J Exp Mar Biol Ecol 426–427: 78–86.

[21] Payne NL, Gillanders BM, Webber DM, Semmens JM (2010) Interpreting diel activity patterns from acoustic telemetry: the need for controls. Mar Ecol Prog Ser 419: 295–301.

[22] Becker A, Whitfield AK, Cowley PD, Järnegren J, Næsje TF (2011) An assessment of the size structure, distribution and behaviour of fish populations within a temporarily closed estuary using dual frequency identification sonar (DIDSON). J Fish Biol 79: 761–775. 21884111 10.1111/j.1095-8649.2011.03057.x

[23] Espinoza M, Farrugia TJ, Webber DM, Smith F, Lowe CG (2011) Testing a new acoustic telemetry technique to quantify long-term, fine-scale movements of aquatic animals. Fish Res 108: 364–371.

[24] Heupel MR, Semmens JM, Hobday AJ (2006) Automated acoustic tracking of aquatic animals: Scales, design and deployment of listening station arrays. Mar Freshw Res 57: 1–13.

[25] Murchie KJ, Cooke SJ, Danylchuk AJ, Suski CD (2011) Estimates of field activity and metabolic rates of bonefish (*Albula vulpes*) in coastal marine habitats using acoustic tri-axial accelerometer transmitters and intermittent-flow respirometry. J Exp Mar Biol Ecol 396: 147–155.

[26] Payne NL, Gillanders BM, Seymour RS, Webber DM, Snelling EP, et al (2011) Accelerometry estimates field metabolic rate in giant Australian cuttlefish *Sepia apama* during breeding. J An Ecol 80: 422–430. 10.1111/j.1365-2656.2010.01758.x 20880022

[27] Griffiths S (2001) Recruitment and growth of juvenile yellowfin bream, *Acanthopagrus australis* Gunther (Sparidae), in an Australian intermittently open estuary. J Appl Ichthy 17: 240–243.

[28] Pease B, Bell J, Burchmore J, Middleton M, Pollard D (1981) The ecology of fish in Botany Bay: Biology of commercially and recreationally valuable species. Sydney: State Pollution Control Commission. BBS 23B. 287 p.

[29] Pollock BR (1982) Movements and migrations of yellowfin bream, *Acanthopagrus australis* (Gunther), in Moreton May, Queensland as determined by tag recoveries. J Fish Biol 20: 245–252.

[30] Gibbs PJ (2001) Monitoring of natural recolonisation of seagrass and transplant success of *Zostera capricorni* at the Sydney Airport third runway in Botany Bay. Sydney: Sydney Airports Corporation. Final Report to Sydney Airports Corporation Limited and Sydney Ports Corporation. 44p.

[31] Haworth R (2002) Changes in mangrove/salt-marsh distribution in the Georges River estuary, Southern Sydney, 1930-1970. Wetlands (Australia) 20: 80–103.

[32] Walsh CT, Reinfelds IV, Gray CA, West RJ, van der Meulen DE, et al (2012) Seasonal residency and movement patterns of two co-occurring catadromous percichthyids within a south-eastern Australian river. Ecol Freshw Fish 21: 145–159.

[33] Payne NL, Gillanders BM, Semmens J (2011) Breeding durations as estimators of adult sex ratios and population size. Oecologia 165: 341–347. 20668884 10.1007/s00442-010-1729-7

[34] Qasem L, Cardew A, Wilson A, Griffiths I, Halsey LG, et al.. (2012) Tri-axial dynamic acceleration as a proxy for animal energy expenditure; Should we be summing values or calculating the vector? PLoS ONE 7. 10.1371/journal.pone.0031187 PMC3281952 22363576

[35] Wilson RP, White CR, Quintana F, Halsey LG, Liebsch N, et al (2006) Moving towards acceleration for estimates of activity-specific metabolic rate in free-living animals: the case of the cormorant. J An Ecol 75: 1081–1090. 10.1111/j.1365-2656.2006.01127.x 16922843

[36] Halsey LG, Green JA, Wilson RP, Frappell PB (2009) Accelerometry to estimate energy expenditure during activity: Best practice with data loggers. Physiol Biochem Zool 82: 396–404. 19018696 10.1086/589815

[37] Barker D, Allan GL, Rowland SJ, Kennedy JD, Pickles JM (2009) A guide to acceptable proceedures and practices for aquaculture and fisheries research, 3rd Edition. Nelson Bay: Primary Industries (Fisheries) Animal Care and Ethics Committee. 52 p.

[38] Butcher PA, Broadhurst MK, Orchard BA, Ellis MT (2010) Using biotelemetry to assess the mortality and behaviour of yellowfin bream (*Acanthopagrus australis*) released with ingested hooks. ICES J Mar Sc 67: 1175–1184.

[39] Zuur AF, Ieno EN, Walker NJ, Saveliev AA, Smith GM (2009) Mixed Effects Models and Extensions in Ecology with R: Springer.

[40] Pinheiro J, Bates D, DebRoy S, Sarkar D, R Development Core Team (2012) nlme: Linear and Nonlinear Mixed Effects Models. R package version 3.1–104.

[41] Bozdogan H (1987) Model selection and Akaike's Information Criterion: The general theory and its analytical extensions. Physcometrika 52: 345–370.

[42] Venables WN, Ripley BD (2002) Modern Applied Statistics with S. New York: Springer. 495 p.

[43] Aitken LS, West SG (1991) Multiple regression: Testing and interpreting interactions. Newbury ParkCalifornia: Sage Publications Inc. 224 p.

[44] Schabenberger O, Pierce FJ (2002) Contemporary Statistical Models for the Plant and Soil Sciences. Baton Rouge: CRC Press. 730 p.

[45] Halle S (2000) Ecological relevance of daily activity patterns. In: Halle S, Stenseth NC, editors. Ecological studies: activity patterns in small mammals, an ecological approach. New York: Springer. pp. 67–90.

[46] Gattermann R, Johnston RE, Yigit N, Fritzsche P, Larimer S, et al (2008) Golden hamsters are nocturnal in captivity but diurnal in nature. Biol Lett 4: 253–255. 18397863 10.1098/rsbl.2008.0066 PMC2610053

[47] de Coursey PJ, Walker JK, Smith SA (2000) A circadian pacemaker in free-living chipmunks: essential for survival? J Comp Physiol A: Sens Neur Behah Physiol 186: 169–180. 10.1007/s003590050017 10707315

[48] Enright JT (1965) Entraintment of a tidal rhythm. Science 147: : 864–&. 10.1126/science.147.3660.864 17793561

[49] Gibson RN (1973) Tidal and circadian activity rhythms in juvenile plaice, *Pleuronectes platessa* . Mar Biol 22: 379–386.

[50] Smith CL, Hill AE, Foreman MG, Peña MA (2001) Horizontal transport of marine organisms resulting from interactions between diel vertical migration and tidal currents off the west coast of Vancouver Island. Can J Fish Aquat Sci 58: 736–748.

[51] Meyer C, Papastamatiou Y, Holland K (2007) Seasonal, diel, and tidal movements of green jobfish (*Aprion virescens*, Lutjanidae) at remote Hawaiian atolls: Implications for marine protected area design. Mar Biol 151: 2133–2143.

[52] Brenner M, Krumme U (2007) Tidal migration and patterns in feeding of the four-eyed fish *Anableps anableps* L. in a north Brazilian mangrove. J Fish Biol 70: 406–427.

[53] Shepard ELC, Ahmed MZ, Southall EJ, Witt MJ, Metcalfe JD, et al (2006) Diel and tidal rhythms in diving behaviour of pelagic sharks identified by signal processing of archival tagging data. Mar Ecol Prog Ser 328: 205–213.

[54] Cummings S, Morgan E (2001) Time keeping system of the eel pout, *Zoarces vivparus* . Chronobiology International 18: 27. 11247112 10.1081/cbi-100001173

[55] Metcalfe JD, Hunter E, Buckley AA (2006) The migratory behaviour of North Sea plaice: Currents, clocks and clues. Marine and Freshwater Behaviour and Physiology 39: 25–36.

[56] Ochwada F, Loneragan N, Gray C, Suthers I, Taylor M (2009) Complexity affects habitat preference and predation mortality in postlarval *Penaeus plebejus*: implications for stock enhancement. Mar Ecol Prog Ser 380: 161–171.

[57] Taylor MD, Fielder DS, Suthers IM (2006) Spatial and ontogenetic variation in the diet of wild and stocked mulloway (*Argyrosomus japonicus*, Sciaenidae) in Australian estuaries. Est Coast 29: 785–793.

[58] Driggers III WB, Campbell MD, Hoffmayer ER, Ingram Jr GW (2012) Feeding chronology of six species of carcharhinid sharks in the western North Atlantic Ocean as inferred from longline capture data. Mar Ecol Prog Ser 465: 185–192.

[59] Ahrens RNM, Walters CJ, Christensen V (2012) Foraging arena theory. Fish and Fisheries 13: 41–59.

[60] Walters C, Christensen V (2007) Adding realism to foraging arena predictions of trophic flow rates in Ecosim ecosystem models: Shared foraging arenas and bout feeding. Ecol Model 209: 342–350.

[61] Kroon FJ (2005) Behavioural avoidance of acidified water by juveniles of four commercial fish and prawn species with migratory life stages. Mar Ecol Prog Ser 285: 193–204.

[62] Thomas BE, Connolly RM (2001) Fish use of subtropical saltmarshes in Queensland, Australia: Relationships with vegetation, water depth and distance onto the marsh. Mar Ecol Prog Ser 209: 275–288.

